# Improved Point-Line Feature Based Visual SLAM Method for Indoor Scenes

**DOI:** 10.3390/s18103559

**Published:** 2018-10-20

**Authors:** Runzhi Wang, Kaichang Di, Wenhui Wan, Yongkang Wang

**Affiliations:** 1 State Key Laboratory of Remote Sensing Science, Institute of Remote Sensing and Digital Earth, Chinese Academy of Sciences, No. 20A, Datun Road, Chaoyang District, Beijing 100101, China; wangrz@radi.ac.cn (R.W.); dikc@radi.ac.cn (K.D.); 2 College of Resources and Environment, University of Chinese Academy of Sciences, Beijing 100049, China; 3 School of Environment Science and Spatial Informatics, China University of Mining and Technology, Xuzhou 221116, China; ts16160030a3@cumt.edu.cn

**Keywords:** indoor visual SLAM, adaptive model, motion estimation, stereo camera

## Abstract

In the study of indoor simultaneous localization and mapping (SLAM) problems using a stereo camera, two types of primary features—point and line segments—have been widely used to calculate the pose of the camera. However, many feature-based SLAM systems are not robust when the camera moves sharply or turns too quickly. In this paper, an improved indoor visual SLAM method to better utilize the advantages of point and line segment features and achieve robust results in difficult environments is proposed. First, point and line segment features are automatically extracted and matched to build two kinds of projection models. Subsequently, for the optimization problem of line segment features, we add minimization of angle observation in addition to the traditional re-projection error of endpoints. Finally, our model of motion estimation, which is adaptive to the motion state of the camera, is applied to build a new combinational Hessian matrix and gradient vector for iterated pose estimation. Furthermore, our proposal has been tested on EuRoC MAV datasets and sequence images captured with our stereo camera. The experimental results demonstrate the effectiveness of our improved point-line feature based visual SLAM method in improving localization accuracy when the camera moves with rapid rotation or violent fluctuation.

## 1. Introduction

Simultaneous localization and mapping (SLAM) is used to incrementally estimate the pose of a moving platform and simultaneously build a map of the surrounding environment [1,2,3]. Owing to its ability of autonomous localization and environmental perception, SLAM has become a key prerequisite for robots to operate autonomously in an unknown environment [4]. Visual SLAM, a system that uses a camera as its data input sensor, is widely used in platforms moving in indoor environments. Compared with radar and other range-finding instruments, a visual sensor has the advantages of low power consumption and small volume, and it can provide more abundant environmental texture information for a moving platform. Consequently, visual SLAM has drawn increasing attention in the research community [5]. As a unique example, integration of visual odometry (VO) with these strategies has been applied successfully to planet rover localization of many planetary exploration missions [6,7,8,9], and has assisted the rovers to travel through challenging planetary surfaces by providing high-precision visual positioning results. Subsequently, many researchers attempted to improve the efficiency and robustness of SLAM methods. In terms of improving efficiency, some feature extraction algorithms such as Speeded-Up Robust Features (SURF) [10], Binary Robust Invariant Scalable Keypoints (BRISK) [11], and oriented FAST and rotated BRIEF (ORB) [12] were proposed. Further, some systems introduced parallel computing to improve efficiency, such as Parallel Tracking and Mapping (PTAM) for small Augmented Reality (AR) workspaces [13]. This is the first SLAM system to separate feature tacking and mapping as two threads, realizing real-time SLAM. As for improving accuracy and robustness, some SLAM systems have introduced the bag-of-words model [14] for the detection of loop closure. Once a loop closure is detected, the closure error is greatly reduced. In recent years, the ability of autonomous localization and environmental perception has rendered visual SLAM an important method, especially in global navigation satellite system (GNSS) denied environments such as indoor scenes [15,16].

Visual SLAM can be implemented using a monocular camera [17,18,19,20], multi-camera [21,22,23], and RGB-D camera [24,25,26] setups. The iterative closet point (ICP) algorithm is used in motion estimation from consecutive frames containing dense point clouds and has been applied effectively in RGB-D-based SLAM [27,28]. However, dense point clouds, produced by dense matching and triangulation of stereo or multi-camera, have uncertainties and invalid regions in environments of low texture and illumination change [29], so in most of the visual SLAM methods, sparse feature extraction and matching are employed to calculate pose of the moving platform. Point and line segments are the two types of primary features used in visual SLAM. Point features have been predominantly used because of their convenient parameterization and implementation in feature tracking between consecutive frames. The visual SLAM systems based on point features estimate camera pose and build an environmental map by minimizing the reprojection error of the observed and corresponding reprojected point features. Furthermore, this optimization process is often solved using the general graph optimization algorithm [30]. ORB-SLAM2 is a representative state-of-the-art visual SLAM method based on point feature [31]; it supports monocular cameras, stereo cameras, and RGB-D cameras, and can produce high-precision results in real time.

In addition to point feature-based visual SLAM systems, line feature-based SLAM systems have been developed recently. Although a line feature is not as easily parameterized as a point feature, as a higher-dimensional feature than a point feature, it can express more environmental information in indoor scenes. Zhang et al. built a graph-based visual SLAM system using 3D straight lines instead of a point feature for localization and mapping [32]. StructSLAM used structure lines of buildings and demonstrated the advantage of a line feature in an indoor scene with many artificial objects [33]. Although the line features can provide more structural information, their endpoints are instable. This problem has been tackled in [34] by utilizing relaxed constraints on their positions.

The above systems use point and line features separately. Some visual SLAM methods combine point and line features. For example, a semi-direct monocular VO, named PL-SVO [35], can obtain more robust results in low-textured scenes by combining points and line segments. The PL-SVO uses the photometric difference between pixels of the same 3D line segment point to estimate the pose increment. The authors of PL-SVO also proposed a robust point-line feature-based stereo VO [36]. In this stereo system, the camera motion is recovered through non-linear minimization of the projection errors of two kinds of features. Based on the work of [35,36], the authors extended [36] with loop closure detection algorithm, and developed a stereo SLAM system named PL-SLAM [37]. Note that there is also a real-time monocular visual SLAM [38], which combines point and line features for localization and mapping, and the nonlinear least square optimization model of point and line features is similar to [37]. The major difference between them is that the former uses a monocular camera and the latter uses a stereo camera. In literature [39], the authors proposed a tightly-coupled monocular visual-inertial odometry (VIO) system exploiting both point-line features and inertial measurement units (IMUs) to estimate the state of camera. In those point-line feature based VO or SLAM systems, the distances from the two re-projected endpoints to the observed line segments are often used as the values to be optimized. However, the structural information of line segments, such as the angle between the re-projected and observed line segments, is not considered in the process of optimization. Furthermore, only the VO system in [36] weighted the errors of different features according to their covariance matrices, and other reported systems do not consider the distribution of weight among different features. Di et al. obtained the inverse of the error as the weights of different data sources in RGB-D SLAM and achieved good results [40], but the motion information of the camera was not considered.

In this paper, an improved point-line feature based visual SLAM method in indoor scenes is proposed. First, unlike the traditional nonlinear least square optimization model of line segment features, an improvement of our method is the addition of the minimization of angle observation, which should be close to zero between the line segments of observation and re-projection. Compared with the traditional nonlinear least square optimization model, which includes distances between the re-projected endpoints and the observed line segment, our method combines angle observation and distance observation and shows a better performance at large turns. Second, our visual SLAM method builds an adaptive model in motion estimation so that the pose estimation model is adaptive to the motion state of a camera. With these two improvements, our visual SLAM can fully utilize point and line segment features irrespective of whether the camera is moving or turning sharply. Experimental results on EuRoC MAV datasets and sequence images captured with our stereo camera are presented to verify the accuracy and effectiveness of this improved point-line feature-based visual SLAM method in indoor scenes.

## 2. Methodology

**Figure 1:**
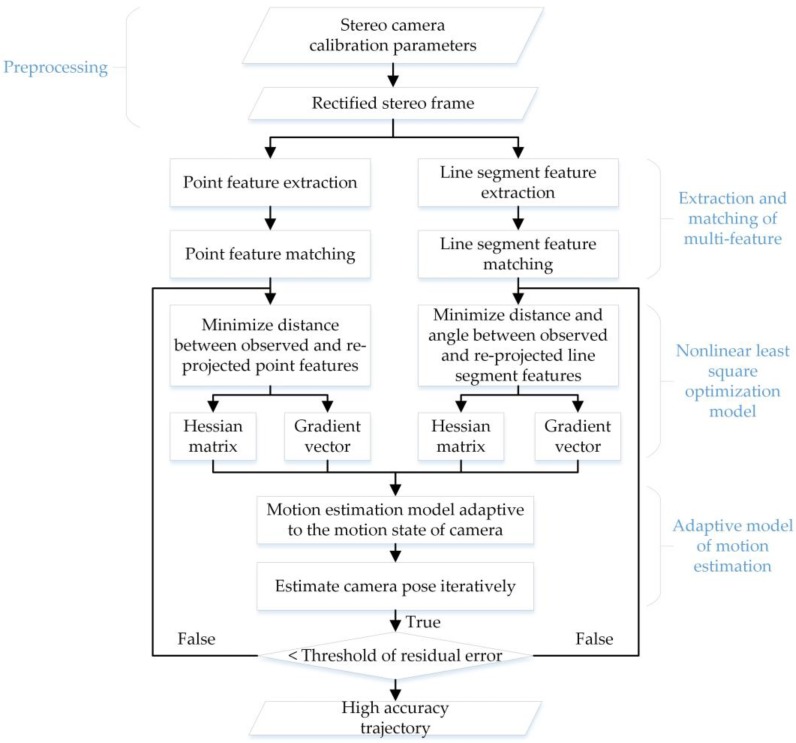
Flowchart of our proposed visual SLAM method.

Figure 1 illustrates our method in a simplified sequence flowchart, which consists of the following main parts: (1) extraction and matching of point and line segment features; (2) building nonlinear least square optimization models of the two kinds of features; (3) motion estimation with an adaptive model. Technical details of the algorithms and models are given in the following sub-sections.

### 2.1. Extraction and Matching of Point and Line Segment Features

**Figure 2:**
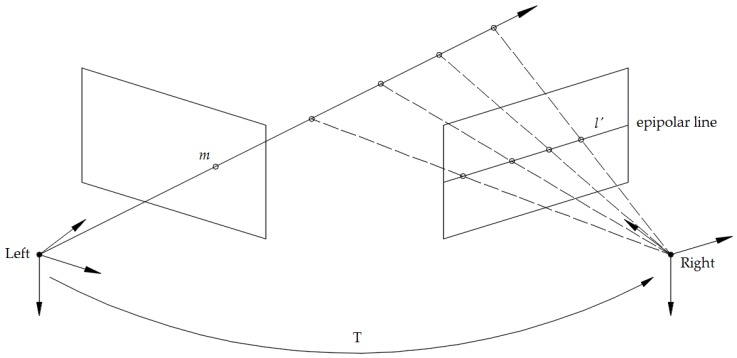
Illustration of the fundamental matrix constraint.

In point feature tracking, the ORB algorithm [12] is adopted in our method to extract 2D point features and create binary descriptors for initial matching. The matching of the extracted point features in consecutive frames is followed by random sample consensus (RANSAC) algorithm and a fundamental matrix constraint, which is used to eliminate some erroneous corresponding keypoints from the matched results. The fundamental matrix constraint is also called epipolar constraint. That is, if the point m of the left image is obtained, its corresponding point on the right image will be constrained on the epipolar line $l^{\prime }$ like Figure 2 shows. As a stereo camera has a baseline, we can calculate the depths and 3D coordinates of all the keypoints with respect to the optical center of the left camera.

**Figure 3:**
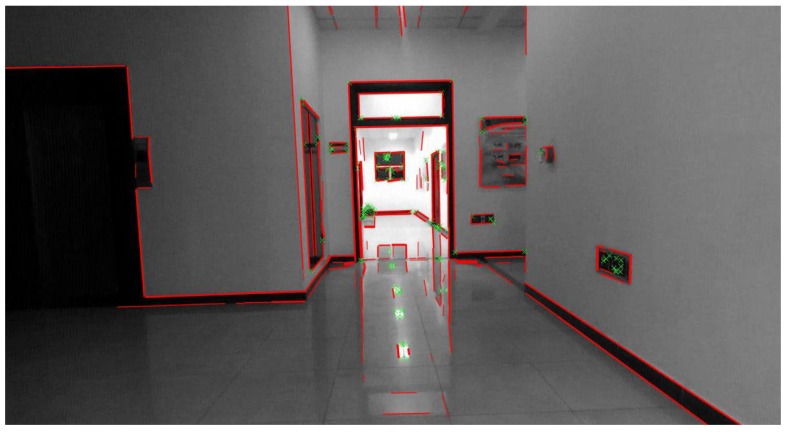
Common low-texture environment in an indoor scene with white walls, and point features (green x marks) extracted using ORB algorithm and line segment features (red lines) extracted using LSD algorithm.

We use the line segment detector (LSD) algorithm [41] for the extraction of line segment features. It can extract line segment features from indoor scenes in linear time, satisfying the real-time requirement of SLAM. Although there are many low-texture environments such as white walls in indoor scenes, the LSD algorithm can stably extract line features, as shown in Figure 3. Furthermore, the line band descriptor method [42] is employed to match line segment features in stereo and consecutive frames with binary descriptors. Similar to the matched point features, we can obtain the 3D coordinates of two endpoints of line segment features and their 2D coordinates.

### 2.2. Nonlinear Least Square Optimization Models for Motion Estimation

Once the 3D and 2D coordinates of the point and line segment features are obtained, the relationships between two consecutive frames can be established using the homologous features. Subsequently, the point and line segment features are back-projected from the previous frame to the current frame. Subsequently, back-projection error models are built for both the points and the line segments. As these error models are nonlinear, the camera motion should be iteratively estimated using the nonlinear least square optimization method. In this study, we use the Gauss–Newton algorithm for the minimization of the back-projection errors. This section presents the nonlinear least square optimization models of point and line segment features.

#### 2.2.1. Optimization Model of Point Features

**Figure 4:**
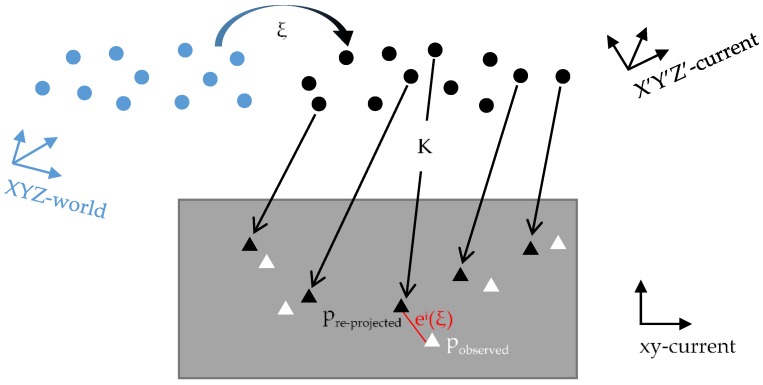
Process of building an error model of point features. The blue dots and black dots represent the world 3D map points and current 3D map points, respectively. The black triangles represent the re-projected 2D points and the white triangles represent the observed 2D points.

For the point features, we use the perspective-n-point method to optimize the camera pose. The error model is the re-projection error, which is the difference between the re-projected 2D position and the observed (matched) 2D position. The optimization process can be divided into three steps. First, 3D map points at the current frame are obtained from stereo image matching and space intersection computation. The world 3D map points are transformed into the coordinate system of the current frame using the iteratively estimated pose of the camera. Subsequently, these 3D map points are re-projected into the image coordinate system of the current frame. Finally, by minimizing the distance error between the re-projected points and their corresponding observed points on the current frame, the error model of point features can be established. This process is illustrated in Figure 4.

In the re-projection error model, the error of the *i*-th point feature can be described as follows:

(1) $$e_{p}^{i}(\zeta )=K\cdot T(\zeta )\cdot P_{XYZ-world}-p.$$

Here, ζ is a six-dimensional vector of Lie algebras that represents the motion of the camera, and T(ζ) represents the transformation matrix from the world coordinate system $P_{XYZ-world}(X,Y,Z)$ to the current coordinate system P′(X′,Y′,Z′) based on the pose of the camera. K represents the internal parameters of the camera and p(x,y) is the corresponding observed point of the re-projected point p′(x′,y′). $e_{p}^{i}(\zeta )$ is the resultant error vector.

To use the Gauss–Newton method, the partial derivative of the error function with respect to the variables is required, which is the Jacobian matrix $\frac{\partial e_{p}^{i}(\zeta )}{\partial \zeta }$ and can be obtained via the chain rule:

(2) $$\frac{\partial e_{p}^{i}(\zeta )}{\partial \zeta }=\frac{\partial e_{p}^{i}(\zeta )}{\partial p\prime }\frac{\partial p\prime }{\partial P\prime }\frac{\partial P\prime }{\partial \zeta }=\frac{\partial e_{p}^{i}(\zeta )}{\partial p\prime }\frac{\partial p\prime }{\partial \zeta }.$$

By calculating $\frac{\partial p\prime }{\partial P\prime }$ and $\frac{\partial P\prime }{\partial \zeta }$, we can obtain $\frac{\partial p\prime }{\partial \zeta }$ as follows:

(3) $$\frac{\partial p\prime }{\partial \zeta }=\left[\begin{array}{cccccc} f_{x}\frac{1}{Z\prime } & 0 & -f_{x}\frac{X\prime }{Z\prime^{2}} & -f_{x}\frac{X\prime Y\prime }{Z\prime^{2}} & f_{x}(1+\frac{X\prime^{2}}{Z\prime^{2}}) & -f_{x}\frac{Y\prime }{Z\prime }\\ 0 & f_{y}\frac{1}{Z\prime } & -f_{y}\frac{Y\prime }{Z\prime^{2}} & -f_{y}(1+\frac{Y\prime^{2}}{Z\prime^{2}}) & f_{y}\frac{X\prime Y\prime }{Z\prime^{2}} & f_{y}\frac{X\prime }{Z\prime } \end{array}\right].$$

As for $\frac{\partial e_{p}^{i}(\zeta )}{\partial p\prime }$, it is a function matrix whose independent variables are the pixel coordinates x′ and y′. Thus, the following equation is obtained:

(4) $$\frac{\partial e_{p}^{i}(\zeta )}{\partial p\prime }=\left[\begin{array}{cc} \frac{\partial (x\prime -x)}{\partial x\prime } & \frac{\partial (x\prime -x)}{\partial y\prime }\\ \frac{\partial (y\prime -y)}{\partial x\prime } & \frac{\partial (y\prime -y)}{\partial y\prime } \end{array}\right]=\left[\begin{array}{cc} 1 & 0\\ 0 & 1 \end{array}\right]=I_{2*2}.$$

After $\frac{\partial e_{p}^{i}(\zeta )}{\partial p\prime }$ and $\frac{\partial p\prime }{\partial \zeta }$ are calculated, we can obtain the Jacobian matrix of point features $\frac{\partial e_{p}^{i}(\zeta )}{\partial \zeta }$. In this study, the Gauss–Newton algorithm is used for the iterative estimation of the camera motion. Therefore, we must calculate the Hessian matrix $H_{p}^{i}$ and gradient vector $g_{p}^{i}$ required by the Gauss–Newton algorithm. The Jacobian matrix is represented by $J_{p}$ and the Hessian matrix and gradient vector can be obtained as follows:

(5) $$\left\{ \begin{array}{l} H_{p}^{i}=J_{p}{}^{T}\cdot P\cdot J_{p}\\ g_{p}^{i}=-J_{p}{}^{T}\cdot P\cdot e_{p}^{i}(\zeta ) \end{array}\right.,$$

where P is the weight matrix of a point feature and can be defined as:

(6) $$P=\left[\begin{array}{cc} \frac{1}{1+\left\|e_{p}^{i}(\zeta )\right\|} & 0\\ 0 & \frac{1}{1+\left\|e_{p}^{i}(\zeta )\right\|} \end{array}\right].$$

Thus, we can add $H_{p}^{i}$ and $g_{p}^{i}$ of each point and obtain the Hessian matrix $H_{p}$ and gradient vector $g_{p}$ of all point features in the current frame:

(7) $$H_{p}=\sum_{i=1}^{n}H_{p}^{i},\;g_{p}=\sum_{i=1}^{n}g_{p}^{i}.$$

Through the above steps, the optimization model of point features is established.

#### 2.2.2. Optimization Model of Line Segment Features

**Figure 5:**
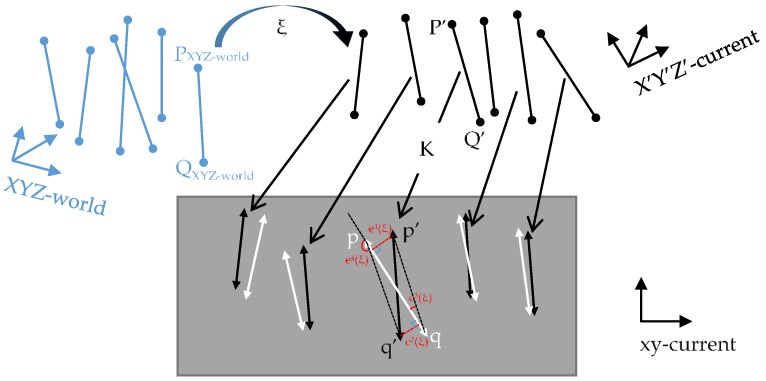
Process of building an error model of line segment features. The blue lines and black lines represent the world 3D map line segments and current 3D map line segments, respectively. The black and white lines with triangular endpoints represent the re-projected and observed line segments, respectively.

As for the error model of line segment features, we use two kinds of error functions. One is the traditional minimization of the distances from the re-projected endpoints to the observed line segment. The other is our proposal for use in this study: the error of angle observation, which should be close to zero between the line segments of observation and re-projection. Each line segment feature has two distance errors and two angle observation errors. The process of establishing an error model of line segment features is shown in Figure 5. Consequently, by minimizing both the distance errors and the angle observation errors, the optimization model of line segment features can be established.

The error function of the *j*-th line segment feature is an 4×1 error vector $e_{l}^{j}(\zeta )$, which can be expressed as follows:

(8) $$e_{l}^{j}(\zeta )=\left[\begin{array}{l} e^{1}(\zeta )\\ e^{2}(\zeta )\\ e^{3}(\zeta )\\ e^{4}(\zeta ) \end{array}\right]=\left[\begin{array}{l} a\times x_{p\prime }+b\times y_{p\prime }+c\\ a\times x_{q\prime }+b\times y_{q\prime }+c\\ (\vec{qp\prime }\cdot \vec{qp})/(\left\|\vec{qp\prime }\right\|\times \left\|\vec{qp}\right\|)-1\\ \left(\vec{pq\prime }\cdot \vec{qp})/(\left\|\vec{pq\prime }\right\|\times \left\|\vec{qp}\right\|)-(-1\right) \end{array}\right],$$

where:

$$\left\{ \begin{array}{c} p\prime (x_{p\prime },y_{p\prime })=K\cdot \exp(\zeta^{\wedge })\cdot P_{XYZ-world}\\ q\prime (x_{q\prime },y_{q\prime })=K\cdot \exp(\zeta^{\wedge })\cdot Q_{XYZ-world} \end{array}\right..$$

In Equation (8), a, b, and c are the three coefficients of the general equation of the observed line. Point $p(x_{p},y_{p})$ and point $q(x_{q},y_{q})$ are the starting and ending endpoints of the observed line, respectively. Similarly, point $p\prime (x_{p\prime },y_{p\prime })$ and point $q\prime (x_{q\prime },y_{q\prime })$ represent the endpoints of the re-projected line segment. The error $e^{1}(\zeta )$ can be considered the distance between point p′ and the observed line pq, and error $e^{2}(\zeta )$ is the distance between point q′ and the observed line. In addition to the two error functions of distance, we add the error functions of angle. Here, $e^{3}(\zeta )$ is the cosine of the angle between vector $\vec{qp\prime }$ and vector $\vec{qp}$, and $e^{4}(\zeta )$ is the cosine of the angle between vector $\vec{pq\prime }$ and vector $\vec{qp}$.

Similar to the point features, we use the chain rule to calculate the Jacobian matrix $\frac{\partial e_{l}^{j}(\zeta )}{\partial \zeta }$ of line segment features as follows:

(9) $$\frac{\partial e_{l}^{j}(\zeta )}{\partial \zeta }=\left[\begin{array}{l} \frac{\partial e^{1}(\zeta )}{\partial p\prime }\frac{\partial p\prime }{\partial P\prime }\frac{\partial P\prime }{\partial \zeta }\\ \frac{\partial e^{2}(\zeta )}{\partial q\prime }\frac{\partial q\prime }{\partial Q\prime }\frac{\partial Q\prime }{\partial \zeta }\\ \frac{\partial e^{3}(\zeta )}{\partial p\prime }\frac{\partial p\prime }{\partial P\prime }\frac{\partial P\prime }{\partial \zeta }\\ \frac{\partial e^{4}(\zeta )}{\partial q\prime }\frac{\partial q\prime }{\partial Q\prime }\frac{\partial Q\prime }{\partial \zeta } \end{array}\right]=\left[\begin{array}{l} \frac{\partial e^{1}(\zeta )}{\partial p\prime }\frac{\partial p\prime }{\partial \zeta }\\ \frac{\partial e^{2}(\zeta )}{\partial q\prime }\frac{\partial q\prime }{\partial \zeta }\\ \frac{\partial e^{3}(\zeta )}{\partial p\prime }\frac{\partial p\prime }{\partial \zeta }\\ \frac{\partial e^{4}(\zeta )}{\partial q\prime }\frac{\partial q\prime }{\partial \zeta } \end{array}\right].$$

The 3D coordinates of the endpoints $P\prime (X_{P\prime },Y_{P\prime },Z_{P\prime })$ and $Q\prime (X_{Q\prime },Y_{Q\prime },Z_{Q\prime })$ are obtained using the pose transformation $\exp(\zeta^{\wedge })$. Using Equation (3), we can calculate $\frac{\partial p\prime }{\partial \zeta }$ and $\frac{\partial q\prime }{\partial \zeta }$. The subsequent step is to calculate $\frac{\partial e^{1}(\zeta )}{\partial p\prime }$, $\frac{\partial e^{2}(\zeta )}{\partial q\prime }$, $\frac{\partial e^{3}(\zeta )}{\partial p\prime }$, and $\frac{\partial e^{4}(\zeta )}{\partial q\prime }$. They are functions of points $p\prime (x_{p\prime },y_{p\prime })$ or $q\prime (x_{q\prime },y_{q\prime })$ and can be calculated as follows:

(10) $$\left\{ \begin{array}{c} \frac{\partial e^{1}(\zeta )}{\partial p\prime }=[a,b]\\ \frac{\partial e^{2}(\zeta )}{\partial q\prime }=[a,b] \end{array}\right.,\;\left\{ \begin{array}{l} \frac{\partial e^{3}(\zeta )}{\partial p\prime }=[f_{p\prime x},f_{p\prime y}]\\ \frac{\partial e^{4}(\zeta )}{\partial q\prime }=[f_{q\prime x},f_{q\prime y}] \end{array}\right.,$$

where a and b are the coefficients of the general equation of the observed line; $f_{p\prime x}$, $f_{p\prime x}$, $f_{p\prime x}$, and $f_{p\prime x}$ are partial derivatives of the coordinates of the re-projected points $p\prime (x_{p\prime },y_{p\prime })$ and $q\prime (x_{q\prime },y_{q\prime })$.

(11) $$\left\{ \begin{array}{c} f_{p\prime x}=\frac{(x_{p}-x_{q})\times \left\|\vec{qp}\right\|\times \left\|\vec{qp\prime }\right\|-((x_{p}-x_{q})\times (x_{p\prime }-x_{q})+(y_{p}-y_{q})\times (y_{p\prime }-y_{q}))\times \left\|\vec{qp}\right\|\times (x_{p\prime }-x_{q})/\left\|\vec{qp\prime }\right\|}{\left\|\vec{qp}\right\|\times \left\|\vec{qp}\right\|\times \left\|\vec{qp\prime }\right\|\times \left\|\vec{qp\prime }\right\|}\\ f_{p\prime y}=\frac{(y_{p}-y_{q})\times \left\|\vec{qp}\right\|\times \left\|\vec{qp\prime }\right\|-((x_{p}-x_{q})\times (x_{p\prime }-x_{q})+(y_{p}-y_{q})\times (y_{p\prime }-y_{q}))\times \left\|\vec{qp}\right\|\times (y_{p\prime }-y_{q})/\left\|\vec{qp\prime }\right\|}{\left\|\vec{qp}\right\|\times \left\|\vec{qp}\right\|\times \left\|\vec{qp\prime }\right\|\times \left\|\vec{qp\prime }\right\|}\\ f_{q\prime x}=\frac{(x_{p}-x_{q})\times \left\|\vec{qp}\right\|\times \left\|\vec{pq\prime }\right\|-((x_{p}-x_{q})\times (x_{q\prime }-x_{p})+(y_{p}-y_{q})\times (y_{q\prime }-y_{p}))\times \left\|\vec{qp}\right\|\times (x_{q\prime }-x_{p})/\left\|\vec{pq\prime }\right\|}{\left\|\vec{qp}\right\|\times \left\|\vec{qp}\right\|\times \left\|\vec{qp\prime }\right\|\times \left\|\vec{qp\prime }\right\|}\\ f_{q\prime y}=\frac{(y_{p}-y_{q})\times \left\|\vec{qp}\right\|\times \left\|\vec{pq\prime }\right\|-((x_{p}-x_{q})\times (x_{q\prime }-x_{p})+(y_{p}-y_{q})\times (y_{q\prime }-y_{p}))\times \left\|\vec{qp}\right\|\times (y_{q\prime }-y_{p})/\left\|\vec{pq\prime }\right\|}{\left\|\vec{qp}\right\|\times \left\|\vec{qp}\right\|\times \left\|\vec{qp\prime }\right\|\times \left\|\vec{qp\prime }\right\|} \end{array}\right.$$

Thus, using Equations (3), (10), and (11), we can obtain the Jacobian matrix $\frac{\partial e_{l}^{j}(\zeta )}{\partial \zeta }$ of the *j*-th line segment features.

Similar to the point features, the Hessian matrix $H_{l}^{j}$ and gradient vector $g_{l}^{j}$ of line segment features are also required by the Gauss–Newton algorithm. The Jacobian matrix is represented by $J_{l}$ and the Hessian matrix and gradient vector can be obtained as follows:

(12) $$\left\{ \begin{array}{l} H_{l}^{j}=J_{l}{}^{T}\cdot P\cdot J_{l}\\ \;g_{l}^{j}=-J_{l}{}^{T}\cdot P\cdot e_{l}^{j}(\zeta ) \end{array}\right.,$$

where P is the weight matrix of the *j*-th line segment feature. As the dimensions of the two distance error functions and the two angle error functions are different, they are weighted in two ways. Thus, P can be defined as:

(13) $$P=\left[\begin{array}{cccc} \frac{1}{1+\left\|e^{1}(\zeta )\right\|} & 0 & 0 & 0\\ 0 & \frac{1}{1+\left\|e^{2}(\zeta )\right\|} & 0 & 0\\ 0 & 0 & \frac{1}{\left\|e^{3}(\zeta )\right\|} & 0\\ 0 & 0 & 0 & \frac{1}{\left\|e^{4}(\zeta )\right\|} \end{array}\right].$$

Subsequently, we add $H_{l}^{j}$ and $g_{l}^{j}$ of each line segment and obtain the Hessian matrix $H_{l}$ and gradient vector $g_{p}$ of all the line segment features in the current frame as follows:

(14) $$H_{l}=\sum_{j=1}^{m}H_{l}^{j},\;g_{l}=\sum_{j=1}^{m}g_{l}^{j}.$$

**Figure 6:**
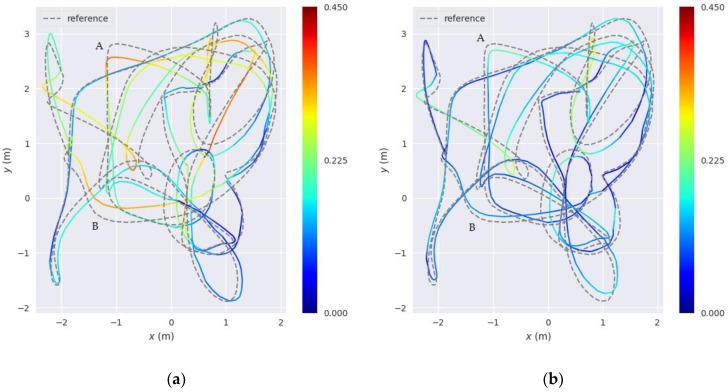
Positioning accuracy heat maps of the two error models. (**a**) The resulting trajectory of the traditional error model used in reference SLAM system; (**b**) The resulting trajectory of the extended error model used in our proposed SLAM system. The gray dotted line represents the ground-truth. The color solid lines represent the accuracy of the trajectories. A change in color from blue to red indicates a gradual increase in the positioning error.

Through these steps, the optimization model of line segment features is established. Compared with the traditional error model of line segment features used in literature [38,39], we add the angular error functions. We have tested our error model on the EuRoC datasets [43] and compare the results with those obtained from the traditional error model. Figure 6 shows the resulting trajectories of the two models tested on dataset Vicon room 1 “medium”. As shown in Figure 6, A and B are two big turns. From the accuracy heat map of the positioning results of these two places, our extended error model with added angular error functions is observed to be superior to the traditional error model.

We use the relative pose error (RPE) as the evaluation metric, which describes the error between pairs of timestamps in the estimated trajectory file. Then we calculate the average RPE at these two big turns A and B to represent the average drift rate between the estimated trajectory and ground-truth. As shown in Table 1, the average RPE of our extended error model is less at A and B than that of traditional error model, meaning that our proposed error model has less drift rate at A and B. This also shows that proposed error model has good robustness at large turns. More detailed results will be given in the experimental results section.

### 2.3. Adaptive Weighting Model of Motion Estimation

After the Hessian matrices and gradient vectors of both point features and line segment features are established, our motion estimation model, which is adaptive to the motion state of a camera, is applied to build a new recombined Hessian matrix and gradient vector for iterated pose estimation.

**Figure 7:**
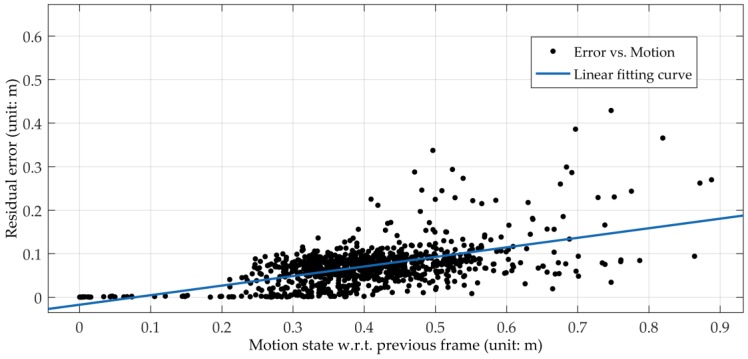
Linear fitting curve between the residual error of the positioning result and the current motion state of a camera. There are 994 motion states and their corresponding residual errors to fit the blue linear curve.

As shown in Figure 7, we have collected the residual errors of nearly 1000 positions and their corresponding motion states of a camera with respect to the previous frame. We use the displacement of the current frame relative to the previous frame as a measure of the current motion state. The blue line in Figure 7 is the linear fitting curve according to these data. It can be observed from Figure 7 that there is a certain degree of correlation between the positioning residual errors and the motion state of the camera. Thus, we calculated the correlation coefficient between them and obtained the result of 0.57. The correlation coefficient is greater than 0.5, indicating that the positioning residual errors and the motion state of the camera are strongly correlated. In other words, the motion state of the camera will affect the positioning result to some extent. However, the reference method does not consider this. Therefore, it can be observed from the accuracy heat maps in the following experiment section that reference method has a large absolute trajectory error (ATE) when the camera moves with large rotation or rapid fluctuation. Hence, we build an adaptive model in the iterative motion estimation. The model is adaptive to the motion state of the camera.

With each iteration, we can obtain the motion of the current frame relative to the previous one. As the frame rate of the camera is fixed, the motion state on the three axes can be represented by the change of camera position ΔP(ΔX,ΔY,ΔZ). If the motion of the camera relative to the previous frame is greater in the image plane direction than in the direction perpendicular to the image plane, i.e., ΔX and ΔY are larger than ΔZ, this indicates that the camera is shaking, which may result in blurred or weakened image texture. According to our experience, line segment features can provide significant structural information of the environment, and hence, the detection of the line segment is more robust than the detection of a point feature in such poor texture scenes. It can also be observed from the experimental results in Figure 6 that the line segment features play an important role when the camera makes a big turn. Thus, in such situations, the weight of the line segment features should be larger than the weight of the point features according to the experimental results and experience. If the motion of the camera relative to the previous frame is greater in the direction perpendicular to the image plane than in the image plane direction, ΔZ will be larger. According to the experiments, the point features in this situation are relatively rich and stable. Hence, the weight of the point features should be larger than the weight of the line segment features. Moreover, we use the inverse of the average re-projection error as a factor in weighting the point feature and line segment feature. Based on the comparative experiments and the above analysis, we propose the following adaptive weighting model of motion estimation:

(15) $$\left\{ \begin{array}{l} W_{p}=\frac{\exp(sqrt({\left(\Delta X\times \mathrm{fps}\right)}^{2}+{\left(\Delta Y\times \mathrm{fps}\right)}^{2}))}{(\sum_{i=1}^{n}\left\|e_{p}^{i}(\zeta )\right\|)/n}\\ W_{l}=\frac{\exp(sqrt({\left(\Delta Z\times \mathrm{fps}\right)}^{2}))}{(\sum_{j=1}^{m}\left\|e_{l}^{j}(\zeta )\right\|)/m} \end{array}\right..$$

With Equation (15), a new recombined Hessian matrix H and gradient vector g can be obtained as follows:

(16) $$\left\{ \begin{array}{c} H=H_{p}\times W_{p}+H_{l}\times W_{l}\\ g=g_{p}\times W_{p}+g_{l}\times W_{l} \end{array}\right..$$

Thus, we can use the Gauss–Newton algorithm to estimate the motion of the camera iteratively. With new frames acquired sequentially, our point-line based visual SLAM system calculates the new positions according to our adaptive weighting model of motion estimation.

## 3. Experimental Results

In this section, to verify the actual performance of the proposed method, we have performed a series of experiments using two types of datasets: public datasets with ground-truth, and sequence images captured using our stereo camera. We also compared our method with the reference method adopted in literature [38,39], which uses the traditional error model of line feature and its weighting model is based on residual errors. All the experiments were performed on a desktop computer with an Intel Core i7-6820HQ CPU with 2.7 GHz and 16 GB RAM without GPU parallelization. The results of the experiments are described in detail below.

### 3.1. EuRoC MAV Datasets

The EuRoC MAV datasets were collected by an on-board micro aerial vehicle (MAV) [43]. They contain two batches of datasets. The first batch was recorded in the Swiss Federal Institute of Technology Zurich (ETH) machine hall and the second batch was recorded in two indoor rooms. They were both captured with a global shutter camera at 20 FPS. Each dataset contains stereo images and accurate ground-truth. Furthermore, calibration parameters such as the intrinsic and extrinsic parameters of the stereo camera are provided in the datasets. We compared our proposed method with the reference method adopted in recent paper and changed the optimization parameters of the reference method to better adapt to different scenarios for fair comparison. We use the absolute trajectory error (ATE) as the evaluation metric, which directly calculates the error between the estimated trajectory and the ground truth [44]. And we calculate both translation and rotation part of ATE as an evaluation of six degree-of-freedom (DoFs).

**Figure 8:**
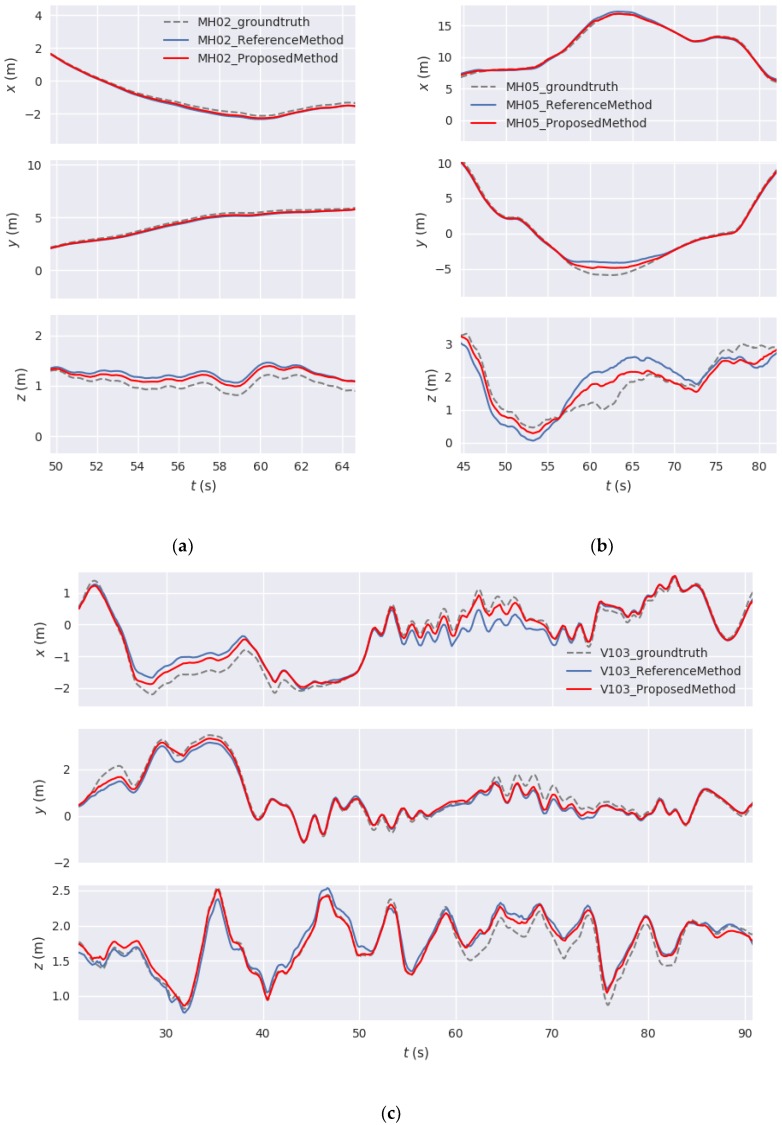
Accuracy of reference method (blue) and our proposed method (red) on the three coordinate axes. (**a**) 50–64 s part of MH_02_easy dataset; (**b**) 45–80 s part of MH_05_difficult dataset. (**c**) 25–90 s part of V1_03_difficult dataset. The trajectories of our proposed method are closer to the ground truth than those of reference method when the camera has large fluctuation.

Figure 8 shows the accuracy of the three coordinate axes on several different datasets. The dotted line represents the ground truth of the dataset. The solid lines in blue and red represent the results of reference method and our proposed method, respectively. As shown in Figure 8a,b, when the Z-axis values have a large fluctuation while the X-axis and Y-axis are stably changing, our proposed method is superior to reference method in the Z-axis. Further, as shown in Figure 8c, when the values of all the three axes fluctuate greatly, our proposed method is more stable and accurate than reference method in these quivering parts. For example, in the X-axis section of Figure 8c from 55–70 s and in the 40–60 s part of the Z-axis, our estimated trajectory is much closer to the ground truth than that of reference method. And then we calculate the average RPE at these places in Figure 8 where camera has rapid fluctuation. The average RPE can represent the average drift rate between the estimated trajectory and ground-truth. As can be seen in Table 2, the average RPE of our proposed method is less than that of reference method, which means our proposed method has less drift rate at these quivering parts.

**Figure 9:**
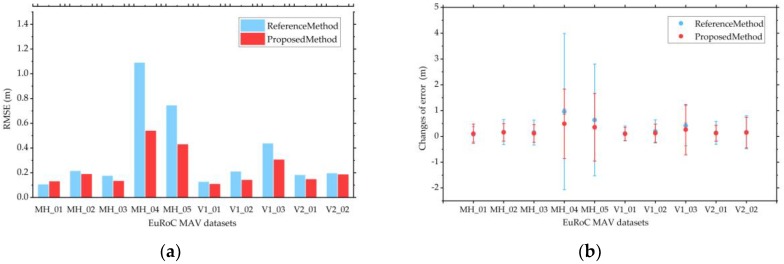
Comparison of RMSEs and the range of translation parts of ATE for reference method and our proposed method using the EuRoC MAV datasets. (**a**) RMSEs of reference method (blue) and our proposed method (red); (**b**) The range of translation parts of ATE of the two methods. The three points on each error bar from top to bottom are the maximum ATE error, average ATE error, and minimum ATE error respectively.

These good performances in the case of rapid fluctuation of camera are mainly attributed to our extended error model and adaptive weighting model of motion estimation. The adaptive weighting model considers both the average re-projection error and the motion state between frames. Therefore, it can better utilize the advantages of different features in different motion states, so as to obtain better positioning results. Figure 8 and Table 2 also confirm that our proposed method performs better when the camera shakes quickly. For quantitative evaluation, we employed the open-source package evo, an easy-to-use evaluation tool (github.com/MichaelGrupp/evo), to evaluate reference method and our proposed method. Table 3 shows the root mean square error (RMSE) of the translation part and rotation part of ATE. Histograms of RMSE and the range of the translation part of ATE are also provided in Figure 9.

Table 3 shows that our proposed method performs better in almost all scenes of EuRoC MAV datasets for the RMSE in terms of the translation parts and rotation parts of ATE. From Figure 9a, in easy and medium scenes, such as MH_02_easy, V1_01_easy, and V2_02_medium, our proposal shows slightly improved accuracy of the results. However, our method can greatly improve the accuracy in difficult scenes, such as MH_04_difficult, MH_05_difficult, and V1_03_difficult. The main reason for such situations is that the camera shakes rapidly in these difficult scenes. Our method considers this situation and better utilizes the respective advantages of point and line segment features through the adaptive weighting model of motion estimation. Furthermore, as shown in Figure 9b, our proposed method has a smaller range of translation parts of ATE, indicating that the motion estimation is relatively stable.

**Figure 10:**
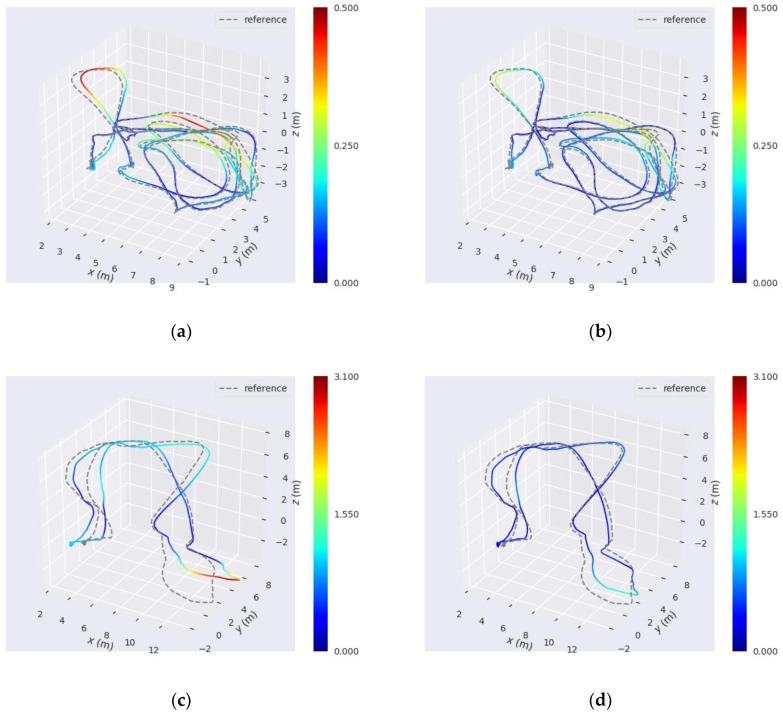

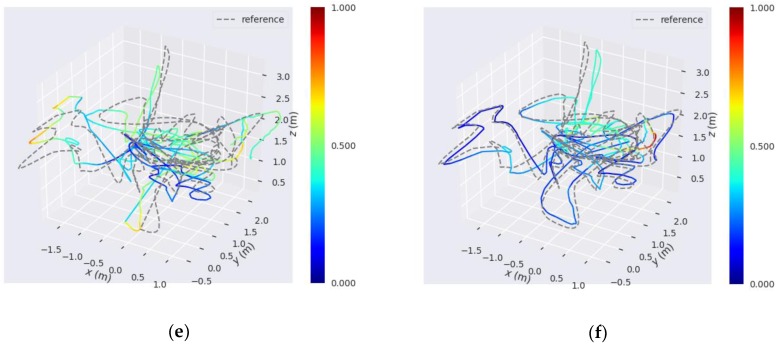
Comparison of several trajectories estimated using reference method and our proposed method. The three accuracy heat maps of the left column are estimated using reference method on the (**a**) MH_03_medium, (**c**) MH_04_difficult, and (**e**) V1_03_difficult sequences. The three accuracy heat maps of the right column are estimated using our proposed method on the (**b**) MH_03_medium, (**d**) MH_04_difficult, and (**f**) V1_03_difficult sequences. The two color bars of each row have the same maximum error and minimum error. The redder the color is, the larger the translation part of ATE is.

To demonstrate the results intuitively, several accuracy heat maps of trajectories estimated using reference method and our proposed method are shown in Figure 10. The gray dotted line represents the ground-truth. The color solid lines represent the estimated trajectories. The color bar represents the size of the translation part of ATE. A change in color from blue to red indicates a gradual increase in translation part of ATE. Each row shows the results of the two methods with the same dataset, and the two color bars of each row have the same maximum error and minimum error. Comparing the three trajectories, we can observe that our method shows better accuracy in some areas with large rotations of camera. This also shows that the angular error function added in our model shows a good performance at large turns. Thus, we can conclude that our proposed method with an adaptive motion model and angular error functions can yield smaller errors than reference method when the camera moves with large rotation or rapid fluctuation.

### 3.2. Sequence Images Captured by Our Stereo Camera

In addition to testing the performance and accuracy of our proposed visual SLAM method on public datasets with ground-truth, we also test the universality with sequence images captured with our stereo camera. The sequence images acquired using the stereo camera should be rectified first in order to use them in high-accuracy SLAM processing. In this experiment, a ZED stereo camera is adopted as our data input sensor. It can capture images with a resolution of 720p at up to 60 fps. Although the ZED camera has been adjusted in production, it does not satisfy the requirements of the experiment. We used Stereo Camera Calibrator, a MATLAB-based software package, to complete the camera calibration process, through which the calibration parameters of the stereo camera including lens distortion coefficients and internal and external parameters were calculated. The calibration results are shown in Table 4 and Table 5. Using these parameters, we can obtain the rectified stereo sequence images.

**Figure 11:**
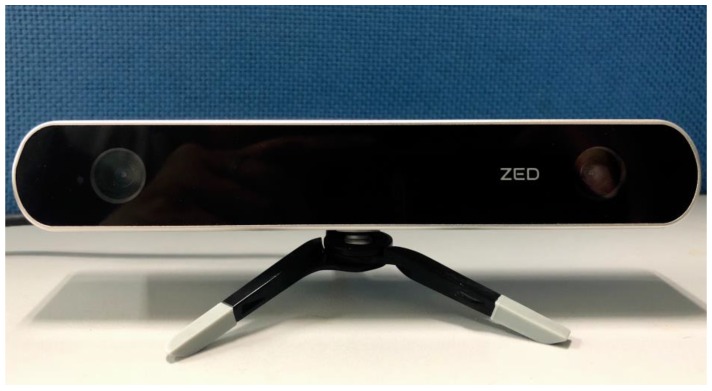
ZED stereo camera used in this experiment.

Figure 11 shows the ZED stereo camera used in this experiment. Figure 3 shows a typical image acquired in this experiment. In the acquisition of sequence images, an operator (one of the co-authors of this paper) first placed the camera at the start point on the floor. Subsequently, he picked up the camera and went on a quadrilateral path along the indoor corridor. Finally, he returned to the starting point. Thus, the whole sequence images form a loop closure. In this experiment, we also present a simple comparison with the point-to-point ICP method adopted in [45]. As no ground truth of the trajectory is available for the sequence images captured with our stereo camera, we evaluate the performance by comparing the closure errors of ICP method, reference method used in before experiment (hereinafter referred to as reference method) and our proposed method. Furthermore, for a fair comparison of the three methods, we do not use loop closure detection in this experiment.

**Figure 12:**
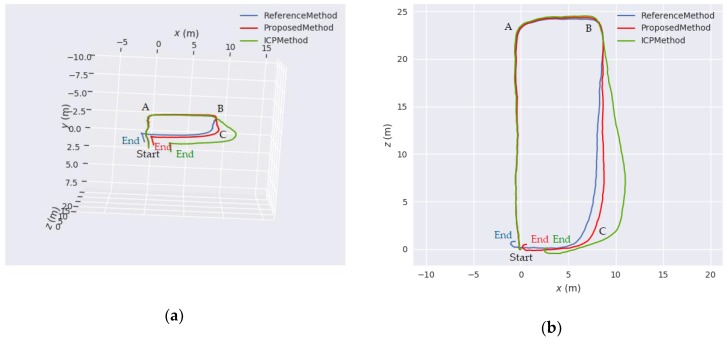
Estimated trajectories from the three methods. The blue curve represent the trajectory estimated using reference method. The red curve represent the trajectory estimated using our proposed method. The green curve represent the trajectory estimated using ICP method. (**a**) 3D display of the three trajectories; (**b**) Top view of the three trajectories.

The statistical results are shown in Table 6 and the three estimated trajectories are shown in Figure 12. The percentage error of our proposed method is 1.07%, which is better than the error obtained using the ICP method, i.e., 4.64%, and also better than the error obtained using the reference method, i.e., 1.82%. The path length calculated by three methods is 64.393 m, 65.272 m and 65.120 m, respectively. The three path lengths are close to each other. However, our method shows only approximately a quarter of the closure error of ICP method and half the closure error of reference method. As observed from the trajectories depicted in Figure 12a, the operator moved from the start point and went forward to corner A. After passing through corner A, he went straight to corner B. We can observe that the trajectories of the three methods are very close to each other in this part. However, from corner B to corner C, the trajectories of ICP method and reference method have a large deviation, which eventually leads to a larger closure error than our method.

By analyzing the motion states at corner A and corner B, we observe that Corner A starts on frame 295 and ends on frame 330 (35 frames in total), whereas Corner B starts on frame 397 and ends on frame 422 (25 frames in total). As the FPS of the camera was fixed, it took more time at corner A. In other words, the speed of the camera at corner B is higher than that at corner A, and hence, the motion is more intense. Further, as shown in Figure 12b, from the top view of the three trajectories, the trajectories of ICP method and reference method begin to deform after corner B. However, owing to the two improvements of our method, i.e., the adaptive weighting model for motion estimation and the angular error functions, the positioning result of our method is less affected by the rapid rotation at corner B than that of ICP method and reference method. Therefore, our trajectory is closer to the predetermined quadrilateral path. This experiment also shows that our proposed method is applicable to the sequence images captured with our stereo camera.

## 4. Conclusions

In this paper, we have presented an improved point-line feature-based visual SLAM method for indoor scenes. The proposed SLAM method has two main innovations: the angular error function added in the optimization process of line segment features, and the adaptive weighting model in iterative pose estimation. Line segment feature is a higher-dimensional feature than point features and has more structural characteristics and geometric constraints. Our optimization model of line segment features with added angular error functions can better utilize this advantage than the traditional optimization model. Furthermore, after the Hessian matrices and gradient vectors of the two kinds of features are established, our model of motion estimation, which is adaptive to the motion state of camera, is applied to build a new recombined Hessian matrix and gradient vector for iterative pose estimation.

We also presented the evaluation results of the proposed SLAM method as compared with the point-line SLAM method developed in [38,39], which uses the traditional error model of line feature and its weighting model is based on residual errors, on both the EuRoC MAV datasets and the sequence images captured with our stereo camera. We also compared the point-to-point ICP method [45] using the sequence images from our stereo camera. According to the experimental results, we arrive at two conclusions. First, the proposed SLAM method has more geometric constraints than the traditional point-line SLAM method and classic ICP method, because the angular error function is added to the optimization model of line segment features. Furthermore, it has good robustness and positioning accuracy at large turns. This is particularly useful for robot navigation in indoor scenes as they include many corners. Second, the adaptive weighting model for motion estimation can better utilize the advantages of point and line segment features in different motion states. Thus, it can improve the system accuracy when the camera moves with rapid rotation or severe fluctuation.

At present, we mainly used the 2D structural constraints of line segment features. In the future, we plan to further improve our SLAM method by introducing the 3D structural constraints of spatial line segment features. Furthermore, topological relations between point features and line segment features will also be considered in our method in the future, so as to better match point and line segment features in indoor environments with repeated textures.

## Figures and Tables

**Table 1:** Average RPE results at A and B turns of traditional error model and our proposed error model. The numbers in bold indicate that these terms are better than those of another model. The unit of RPE is meter.

Turns in Figure 6	Traditional Error Model	Proposed Error Model
A	0.149500	**0.138527**
B	0.142878	**0.131200**

**Table 2:** Average RPE results at three quivering parts of reference method and our proposed method. The numbers in bold indicate that these terms are better than those of another method. The unit of RPE is meter.

Part of Datasets	Reference Method	Proposed Method
MH02 50–64 s	0.086233	**0.084906**
MH05 45–80 s	0.224745	**0.152861**
V103 25–90 s	0.182875	**0.113256**

**Table 3:** Translation parts and rotation parts of ATE of the two methods on several EuRoC MAV datasets. The numbers in bold indicate that these terms are better than those of another method. The unit of translation part is meter and the unit of rotation part is degree.

EuRoC MAV Datasets	Reference Method		Proposed Method	
	ATE (Tran.)	ATE (Rot.)	ATE (Tran.)	ATE (Rot.)
MH_01_easy	**0.103648**	2.642111	0.127967	**1.545874**
MH_02_easy	0.211978	1.367287	**0.187721**	**1.180007**
MH_03_medium	0.173194	1.406898	**0.131740**	**0.861475**
MH_04_difficult	1.088483	5.847468	**0.538066**	**3.039995**
MH_05_difficult	0.742775	6.197293	**0.428191**	**3.260860**
V1_01_easy	0.124277	1.522630	**0.106866**	**1.057048**
V1_02_medium	0.208179	2.397838	**0.139928**	**1.747601**
V1_03_difficult	0.434853	2.777067	**0.304662**	**2.514817**
V2_01_easy	0.179175	2.817506	**0.145582**	**2.487347**
V2_02_medium	0.193462	4.059261	**0.183889**	**3.992289**

**Table 4:** Internal parameters of the left and right camera. The units of $f_{x}$, $f_{y}$, $c_{x}$, and $c_{y}$ are pixels.

Camera	$f_{x}$	$f_{y}$	$c_{x}$	$c_{y}$	$k_{1}$	$k_{2}$	$k_{3}$	$p_{1}$	$p_{2}$
Left Camera	659.38	659.51	605.17	375.78	0.00093	−0.00041	0.0	0.0016	0.00036
Right Camera	659.46	659.52	605.98	375.27	0.00063	0.000037	0.0	0.0013	0.00084

**Table 5:** External parameters of the left camera and right camera.

**Rotation Angles (°)**	0.00015	−0.0012	−0.00077
**Translation Vector (mm)**	−119.7164	0.0348	−0.22480

**Table 6:** Localization results of ICP method, reference method and our proposed method.

Method	Total Length (m)	Closure Error (m)	Percentage Error
ICP Method	64.393	2.9913	4.64%
Reference Method	65.272	1.1912	1.82%
Proposed Method	65.120	0.6988	1.07%

## References

[1] Montemerlo M., Thrun S., Koller D., Wegbreit B. FastSLAM: A factored solution to the simultaneous localization and mapping problem. Proceedings of the 18th National Conference on Artificial Intelligence.

[2] Durrant-Whyte H., Bailey T. (2006). Simultaneous localization and mapping: Part I. IEEE Robot. Autom. Mag..

[3] Bailey T., Durrant-Whyte H. (2006). Simultaneous localization and mapping (SLAM): Part II. IEEE Robot. Autom. Mag..

[4] Dissanayake M.W.M.G., Newman P., Clark S., Durrant-Whyte H.F., Csorba M.A. (2001). Solution to the simultaneous localization and map building (SLAM) problem. IEEE Trans. Robot. Autom..

[5] Fuentes-Pacheco J., Ruiz-Ascencio J., Rendón-Mancha J.M. (2015). Visual simultaneous localization and mapping: A survey. Artif. Intell. Rev..

[6] Cheng Y., Maimone M.W., Matthies L. (2006). Visual odometry on the Mars exploration rovers—A tool to ensure accurate driving and science imaging. IEEE Robot. Autom. Mag..

[7] Maimone M., Cheng Y., Matthies L. (2007). Two years of visual odometry on the mars exploration rovers: Field reports. J. Field Robot..

[8] Di K., Xu F., Wang J., Agarwal S., Brodyagina E., Li R., Matthies L. (2008). Photogrammetric processing of rover imagery of the 2003 Mars Exploration Rover mission. ISPRS J. Photogramm. Remote Sens..

[9] Wang B.F., Zhou J.L., Tang G.S. (2014). Research on visual localization method of lunar rover. Sci. China Inf. Sci..

[10] Bay H., Ess A., Tuytelaars T., Gool L.V. (2008). Speeded-Up Robust Features (SURF). Comput. Vis. Image Underst..

[11] Leutenegger S., Chli M., Siegwart R. BRISK: Binary Robust Invariant Scalable Keypoints. Proceedings of the 2011 IEEE International Conference on Computer Vision (ICCV).

[12] Rublee E., Rabaud V., Konolige K. ORB: An efficient alternative to SIFT or SURF. Proceedings of the 2011 IEEE International Conference on Computer Vision (ICCV).

[13] Klein G., Murray D. Parallel tracking and mapping for small AR workspaces (PTAM). Proceedings of the 2007 6th IEEE and ACM International Symposium on Mixed and Augmented Reality.

[14] Galvez-López D., Tardos J.D. (2012). Bags of binary words for fast place recognition in image sequences. IEEE Trans. Robot..

[15] Ido J., Shimizu Y., Matsumoto Y., Ogasawara T. (2009). Indoor Navigation for a Humanoid Robot Using a View Sequence. Int. J. Robot. Res..

[16] Celik K., Chung S.J., Clausman M., Somani A.K. Monocular vision SLAM for indoor aerial vehicles. Proceedings of the 2009 IEEE/RSJ International Conference on Intelligent Robots and Systems (ICRA).

[17] Davison A.J., Reid I.D., Molton N.D., Stasse O. (2007). MonoSLAM: Real-time single camera SLAM. IEEE Trans. Pattern Anal. Mach. Intell..

[18] Wu K., Di K., Sun X., Wan W., Liu Z. (2014). Enhanced monocular visual odometry integrated with laser distance meter for astronaut navigation. Sensors.

[19] Lemaire T., Lacroix S. Monocular-vision based SLAM using Line Segments. Proceedings of the IEEE International Conference on Robotics and Automation (ICRA).

[20] Celik K., Chung S.J., Somani A. Mono-vision corner SLAM for indoor navigation. Proceedings of the 2008 IEEE International Conference on Electro/information Technology.

[21] Zou D., Tan P. (2013). CoSLAM: Collaborative visual SLAM in dynamic environments. IEEE Trans. Pattern Anal. Mach. Intell..

[22] Moratuwage D., Wang D., Rao A., Senarathne N. (2014). RFS Collaborative Multivehicle SLAM: SLAM in Dynamic High-Clutter Environments. IEEE Robot. Autom. Mag..

[23] Kaess M., Dellaert F. (2010). Probabilistic structure matching for visual SLAM with a multi-camera rig. Comput. Vis. Image Understand..

[24] Hu G., Huang S., Zhao L., Alempijevic A., Dissanayake G. A robust RGB-D SLAM algorithm. Proceedings of the 2012 IEEE/RSJ International Conference on Intelligent Robots and Systems (IROS).

[25] Kerl C., Sturm J., Cremers D. Dense visual SLAM for RGB-D cameras. Proceedings of the 2013 IEEE/RSJ International Conference on Intelligent Robots and Systems.

[26] Ji Y., Yamashita A., Asama H. (2015). RGB-D SLAM using vanishing point and door plate information in corridor environment. Intell. Serv. Robot..

[27] Kim D.H., Kim J.H. (2013). Image-Based ICP algorithm for visual odometry using a RGB-D sensor in a dynamic environment. Adv. Intell. Syst. Comput..

[28] Steinbrücker F., Sturm J., Cremers D. Real-time visual odometry from dense RGB-D images. Proceedings of the 2011 IEEE International Conference on Computer Vision Workshops (ICCV Workshops).

[29] Jiang Y., Chen H., Xiong G., Scaramuzza D. ICP Stereo Visual Odometry for Wheeled Vehicles based on a 1DOF Motion Prior. Proceedings of the 2014 IEEE International Conference on Robotics and Automation (ICRA).

[30] Kümmerle R., Grisetti G., Strasdat H., Konolige K., Burgard W. G2o: A general framework for graph optimization. Proceedings of the 2011 IEEE International Conference on Robotics and Automation (ICRA).

[31] Mur-Artal R., Tardós J.D. (2017). ORB-SLAM2: An Open-Source SLAM System for Monocular, Stereo, and RGB-D Cameras. IEEE Trans. Robot..

[32] Zhang G., Jin H.L., Lim J., Suh I.H. (2015). Building a 3-d line-based map using stereo SLAM. IEEE Trans. Robot..

[33] Zhou H., Zou D., Pei L., Ying R., Liu P., Yu W. (2015). StructSLAM: Visual SLAM with building structure lines. IEEE Trans. Veh. Technol..

[34] Micusik B., Wildenauer H. Structure from Motion with Line Segments under Relaxed Endpoint Constraints. Proceedings of the 2014 International Conference on 3d Vision.

[35] Gomez-Ojeda R., Briales J., Gonzalez-Jimenez J. PL-SVO: Semi-direct Monocular Visual Odometry by combining points and line segments. Proceedings of the 2016 IEEE/RSJ International Conference on Intelligent Robots and Systems (IROS).

[36] Gomez-Ojeda R., Gonzalez-Jimenez J. Robust stereo visual odometry through a probabilistic combination of points and line segments. Proceedings of the 2016 IEEE International Conference on Robotics and Automation (ICRA).

[37] Gomez-Ojeda R., Moreno F.A., Scaramuzza D., Gonzalez-Jimenez J. (2017). PL-SLAM: A Stereo SLAM System through the Combination of Points and Line Segments. arXiv.

[38] Pumarola A., Vakhitov A., Agudo A., Sanfeliu A., Moreno-Noguer F. PL-SLAM: Real-time monocular visual SLAM with points and lines. Proceedings of the 2017 IEEE International Conference on Robotics and Automation (ICRA).

[39] He Y., Zhao J., Guo Y., He W., Yuan K. (2018). PL-VIO: Tightly-Coupled Monocular Visual-Inertial Odometry Using Point and Line Features. Sensors.

[40] Di K., Zhao Q., Wan W., Wang Y., Gao Y. (2016). RGB-D SLAM based on extended bundle adjustment with 2D and 3D information. Sensors.

[41] Grompone V.G.R., Jakubowicz J., Morel J.M., Randall G. (2010). LSD: A fast line segment detector with a false detection control. IEEE Trans. Pattern Anal. Mach. Intell..

[42] Zhang L., Koch R. (2013). An efficient and robust line segment matching approach based on LBD descriptor and pairwise geometric consistency. J. Vis. Commun. Image Represent..

[43] Burri M., Nikolic J., Gohl P., Schneider T., Rehder J., Omari S., Achtelik M.W., Siegwart R. (2016). The EuRoC micro aerial vehicle datasets. Int. J. Robot. Res..

[44] Sturm J., Engelhard N., Endres F., Burgard W., Cremers D. A benchmark for the evaluation of RGB-D SLAM systems. Proceedings of the 2012 IEEE/RSJ International Conference on Intelligent Robots and Systems (IROS).

[45] Milella A., Siegwart R. Stereo-based ego-motion estimation using pixel tracking and iterative closest point. Proceedings of the 2006 IEEE International Conference on Computer Vision Systems.

